# Serviceability and Flexural Behavior of Concrete Beams Reinforced with Basalt Fiber-Reinforced Polymer (BFRP) Bars Exposed to Harsh Conditions

**DOI:** 10.3390/polym12092110

**Published:** 2020-09-16

**Authors:** Hakem Alkhraisha, Haya Mhanna, Noor Tello, Farid Abed

**Affiliations:** Department of Civil Engineering, American University of Sharjah, Sharjah 26666, UAE; b00079649@aus.edu (H.A.); g00023682@alumni.aus.edu (H.M.); g00062985@alumni.aus.edu (N.T.)

**Keywords:** BFRP, exposure, flexure, crack width, bond-dependent coefficient, finite element

## Abstract

The main objective of this study was to investigate experimentally and numerically the behavior of basalt fiber-reinforced polymer (BFRP) reinforcement exposed to a combination of ultraviolet rays, humidity, and rain. Specifically, the effects of the previously stated harsh exposure on the serviceability performance and flexural capacity of BFRP reinforced concrete beams was examined. Holding the exposure parameter constant, the study also evaluated the effects of reinforcement ratio and beam detailing on the flexural capacity and the bond-dependent coefficient (*k_b_*) of the beams. Seven beams were cast and tested, four of which were reinforced with exposed BFRP bars, two were reinforced with unexposed BFRP bars, and one specimen was cast and reinforced with steel bars to serve as a benchmark specimen. The results indicate that the *k_b_* factor was averaged to be 0.61 for all the beams. Test results also indicate that increasing the reinforcement ratio did not result in a directly proportional increase in the moment capacity. The period of exposure did not cause any significant impact on the behavior of the over-reinforced beams. Thus, a finite element model was created to simulate the impact of exposure on the behavior of under-reinforced BFRP reinforced concrete beams.

## 1. Introduction

Fiber reinforced polymer (FRP) reinforcement was investigated as a substitute to the conventional steel reinforcement. FRP reinforcement combats a few of the shortcomings of steel reinforcement. Particularly, FRP reinforcements are known for their high corrosion resistance due to their non-metallic nature [1]. In addition, FRP composites provide higher tensile strength than steel products do [1,2]. Depending on the type of fiber used, FRP composites are categorized as carbon FRP (CFRP), glass FRP (GFRP), basalt FRP (BFRP), and aramid FRP (AFRP) [1,2,3,4]. The aforementioned FRP products differ in properties due to the difference in fiber type, fiber volume, fiber orientation, resin type, chemical composition, and quality control during the development process [1]. The chemical composition of FRP makes them anisotropic, i.e., they show linear elastic behavior until rupture [1,2]. Due to the difference in behavior between steel and FRP composites, the conventional design philosophies, followed in ACI 318, had to be tailored to the new development. The advancement in the realm of FRP led to the birth of a new manual for FRP reinforced concrete design (ACI 440). The manual is still a work in progress, especially in areas such as bond dependent coefficient, BFRP reinforcement, and exposure. This paper attempts to fill in some of the gaps in knowledge by investigating bond dependent coefficient of concrete beams reinforced with BFRP bars exposed to harsh conditions.

Few studies investigated the mechanical behavior [5], compression behavior [6], shear behavior [7,8,9,10], and flexural behavior [11,12,13,14,15] of BFRP reinforcement. Elgabbas et al. [11] studied the behavior of FRP reinforced concrete beams subjected static loading. The study indicated that an increase in reinforcement ratio led to a nonlinear increase in the flexural capacity of FRP reinforced concrete beams. Another study that attempted to investigate the effect of reinforcement ratio on the behavior of FRP reinforced concrete beams was conducted by Fam and Tomlinson [12]. In the study, nine FRP reinforced beams (reinforcement ratio ranging from 0.28 to 1.60) were tested. The results indicate that ultimate and service loads were proportionally affected by the reinforcement ratio.

Although BFRP research in itself is scarce, research on the effect of exposure on BFRP bars is more infrequent. Only few researchers investigated the effects of exposure on the flexural behavior of BFRP reinforced concrete (RC) beams and on the bond behavior between BFRP bars and surrounding concrete. The studies had different areas of focus; while some examined the mechanical behavior of exposed FRP bars, others investigated the effects of exposure on FRP RC beams [16]. The type of exposure tested also varied; alkaline solutions exposure [17,18,19,20], temperature exposure [21,22,23,24,25], and saline solutions exposure [26] were all investigated.

Wu et al. [17] studied the effects of alkaline solution, acid solution, and salt solution on the tensile strength of BFRP bars. The degradation of BFRP bars due to such exposure was monitored using scanning electronic microscopy (SEM). The results show that the acid solution and the salt solution had lesser impact on the durability of BFRP bars than the alkaline solution [17]. Another study that examined the impact of alkali solution, combined with high temperatures, on the durability of FRP bars was conducted by Sim et al. [18]. The results of the study indicate that, after seven-day exposure, BFRP and GFRP bars lost 50% of their volume and strength. CFRP, however, lost only 13% of its strength and volume during the same exposure period. The study also showed that the fiber and resin type played a big role in resisting degradation. Wang et al. [19] also investigated the impact of alkali solution on the durability of BFRP [19]. They found that, after three months of exposure to alkali solution, the strength BFRP decreased by 40% while the modulus of elasticity remained unaffected. The results also indicate that BFRP bars had lower degradation than the basalt fiber with no resin protection [19].

One of the studies that focused on the impact exposure on the bond behavior rather than on the mechanical properties was conducted by Altalmas et al. [26]. They investigated the impact of accelerated aging conditions on the bond degradation of BFRP bars compared with GFRP bars. The bars were exposed to harsh environments such as alkaline, acid, and saline solutions. The results show that, for all exposure types, sand coated BFRP bars possessed higher bond strengths than the ribbed GFRP did. Slippage resistance and bond strength were controlled by surface texture. The study concluded that, generally, sand coated bars showed more resistance than ribbed FRP bars [26].

In general, there are considerable gaps in knowledge regarding topics related to BFRP, especially the impact of exposure on BFRP bars. This study aimed to add to the body of knowledge in the area of exposure impact on the bond strength and flexural behavior of BFRP reinforced concrete beams. The uniqueness of this study is attributed to the uniqueness of the exposure and tests used to measure such exposure. The study enveloped three unique parameters; BFRP behavior is, in itself, the frontier of FRP research, the exposure to duplicate practical site-like exposure, and the effect of exposure, in general, on the serviceability and flexural behavior of BFRP reinforced concrete beams has never been investigated before. The BFRP RC beams were studied as a whole system to see the impact of the exposure on the serviceability of the beams including cracks width and propagation and the bond response.

The parameters of the study were reinforcement ratio, exposure, and beam detailing. The studied properties were flexural capacity, deflection and cracking behavior, bond behavior, and strain of reinforcement and concrete. Experimental results of moments and bond dependent coefficient (*k_b_*) were utilized to assess the design equations of the ACI 440.1R code. To test the impact of exposure on under-reinforced BFRP RC beams, parametric study was conducted using Finite Element Modeling (FEM) software (ABAQUS) [27]. Other research has investigated the behavior of FRP reinforced concrete using FEM [28,29,30,31] but little research has been dedicated to investigating the effects of exposure on under-reinforced BFRP RC beams. Some of the results of this study are briefly analyzed and reported in [32]. The reported results, however, are limited to four beams and the analysis is limited to the flexural behavior.

## 2. Materials and Methods

### 2.1. Material Properties

The sought compressive strength of the concrete mix was 30 MPa. After testing a number of cubes and cylinders obtained from the same mix and exposed to the same curing conditions as the test specimen, the actual compressive strength was found to be 36.5 MPa for cubes and 30.2 MPa for cylinders. All tested specimens were casted from the same concrete batch.

**Figure 1:**
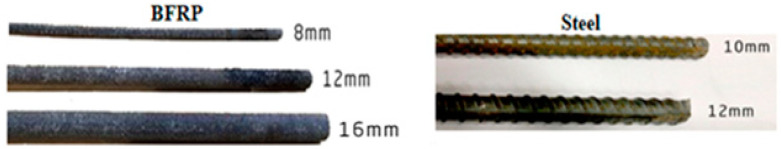
Bars types and sizes utilized.

The reinforcing BFRP bars used in this study were sand coated with 8-, 12-, and 16-mm diameters. In addition, steel rebar with diameter of 12 mm was used for control purposes. The utilized bars are shown in Figure 1. The BFRP bars were supplied from Galen, a Russian manufacturing company based in the city of Cheboksary. Thus, the results of this study can only be generalized for BFRP bars of the same properties and manufacturer processes as provide by Galen. However, the findings are expected to be close as basalt obtained from different locations may exhibit slight variations. The mechanical properties for the bars, as per the manufacturer specifications, are shown in Table 1.

### 2.2. Specimen Configuration and Test Matrix

**Figure 2:**
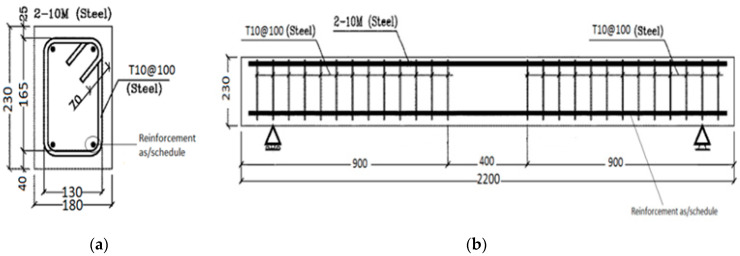
Beam details: (**a**) cross section; and (**b**) elevation view.

Beams were designed in accordance with the ACI 440.1R-15 code [1] with respect to bar spacing, clear cover, and minimum required depth. Furthermore, to ensure the slenderness of the specimens, a/d ≥ 3) span-to-depth was taken into consideration. The resulted cross section is 180 mm × 230 mm, as shown in Figure 2a. The beams were 2200 mm long with a clear span of 1900 mm. Steels stirrups with diameter of 10 mm were utilized at a 100 mm spacing to prevent shear failure. The beam elevation view is shown in Figure 2b.

Seven beams were utilized in this study. As per schedule, 2ϕ8 exposed, 2ϕ12 exposed, 2ϕ16 exposed, 3ϕ12 exposed, 2ϕ8 unexposed, and 2ϕ16 unexposed were used for testing the effects of exposure on BFRP bars. One steel beam consisting of 2ϕ12 steel bars was tested for control purposes. All BFRP reinforced concrete beams were designed to be over-reinforced with *ρ_f_*/*ρ_fb_* > 1.4 while the steel (control) beam was designed to be tension-controlled. The test matrix is shown in Table 2.

### 2.3. Instrumentation and Test Setup

**Figure 3:**
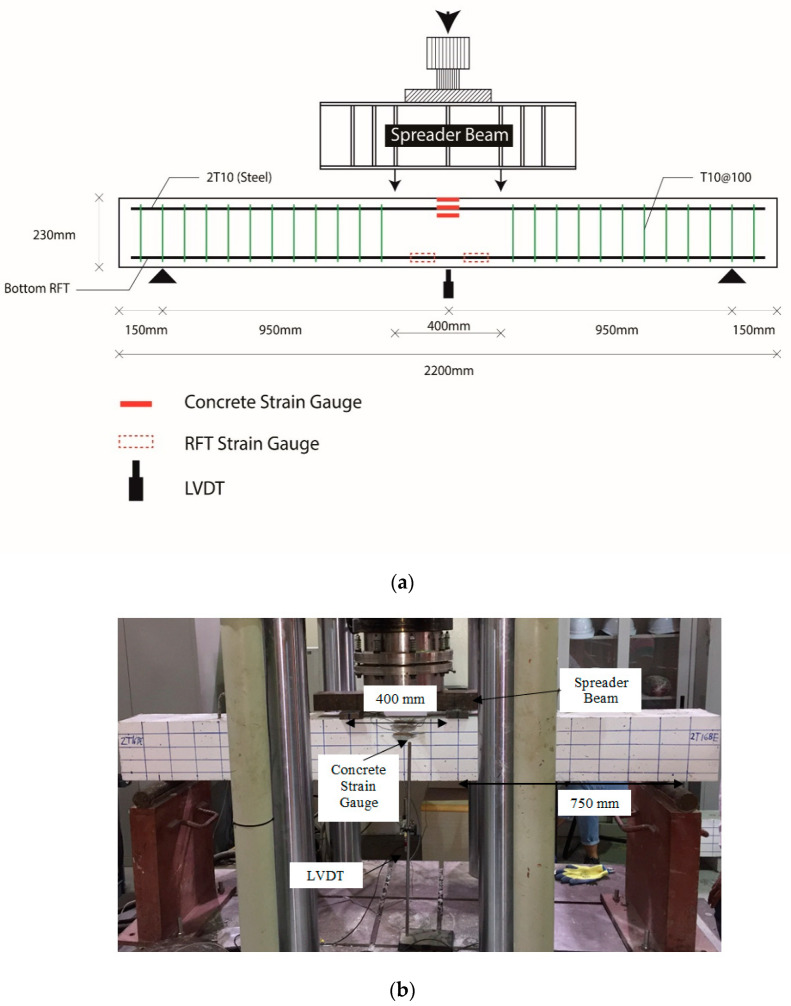
Test configuration: (**a**) instrumentation; and (**b**) test setup.

The four-point loading setup (see Figure 3) was used in this study to analyze the flexural response of all beams. The testing was conducted using an Instron universal testing machine (UTM) (Sigma Enterprises LLC., Dubai, UAE) where jacks were used to apply the load directly on a spreader beam using displacement control mode rate of 1 mm per minute. The spreader beam divides the load equally on two points that are 400 mm apart, which becomes a constant maximum moment region. Strain gauges were fitted on the surface of the concrete beams and on the bottom reinforcing bars to record the strain throughout the test. A variable differential transformer (LVDT) (RDP Group, Dubai, UAE) was installed below the center of the beam to measure deflection. Crack transducers were utilized to measure the crack width during the tests; the crack width was later used to calculate the *k_b_* factors for the tested beams. The schematic of the test setup is shown in Figure 3a and the actual test setup is shown in Figure 3b.

### 2.4. Exposure

**Figure 4:**
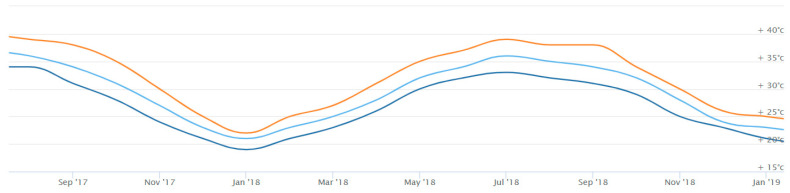
Temperature records in the city of Sharjah (SEP17-JAN19) [33].

**Figure 5:**
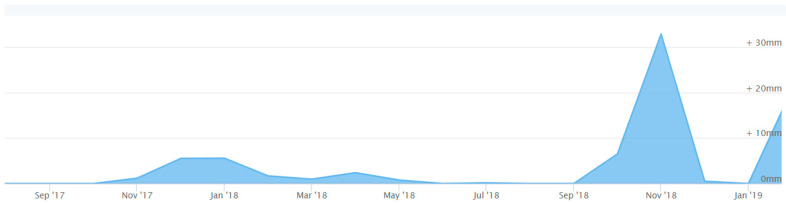
Rainfall records in the city of Sharjah (SEP17-JAN19) [33].

**Figure 6:**
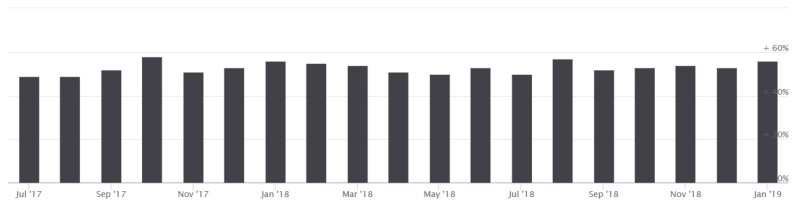
Humidity records in the city of Sharjah (SEP17-JAN19) [33].

The type of exposure, as previously stated, is a unique one. The purpose of the exposure program was to simulate real life conditions in the UAE industry. Bars were left outdoors exposed to the natural weather conditions including temperature, rainfall, and humidity. The exposure period was 15 months, and the location was the city of Sharjah, UAE. The exposure conditions can be generalized to all humid and hot environments. Figure 4 shows the data of temperature during the exposure period, Figure 5 shows the data of rainfall during the exposure period, and Figure 6 shows the data of humidity during the exposure period.

Temperature fluctuates during the year but keeps a constant trend across the years. The temperature peaks during the summer months reaching values of +40 °C and is lowest during the winter months where it drops to just below 20 °C, as shown in Figure 4. The reported temperature is the average temperature of daytime and nighttime. The UAE temperature at noon reaches up to 50 °C, which is still less than the glass transition temperature of 65–120 °C, as reported by the ACI440.1R code [1]. This type of exposure applies only to high temperature is strictly applicable to regions with all year high temperature. To investigate the impact of environments with cold temperatures, other studies must be conducted.

Rain is scarce in Sharjah but nonetheless the rainfall records are reported since the FRP bars were exposed to rain. Throughout the entire duration of the exposure, the largest amount of rainfall recorded was just above 30 mm, which was during the second half of 2018. Rainfall records are included for reference despite the predicted lack of impact on the properties of the FRP bars. Since the bars were outside, the rain records are reported even if rainfall was not the intended type of exposure for the FRP specimen. The reported yearly rainfall for the exposure period is shown in Figure 5.

Humidity fluctuates during the year but keeps a constant trend across the years. The humidity ranged from 50% to 60% during the exposure period, as shown in Figure 6. High humidity can be found across all cities that are exposed to high temperatures and are near large bodies of water. The results of the findings can only be generalized to areas with high temperatures and high humidity.

## 3. Results and Discussion

### 3.1. Cracking Moment

The load causing the first crack was recorded during the testing process. Since the FRP reinforced beams were designed to fail in concrete crushing, the reinforcement ratio did not affect the behavior of the beam prior to the first crack. Concrete strength was the main factor that affected the cracking moment (*M_cr_*). All beams were cast using the same concrete; thus, the predicted cracking moments were the same. The predicted ultimate moment (*M_n_*) differed based on the reinforcement ratio. A summary of the predicted ultimate and cracking moments compared with the experimental results is shown in Table 3.

Table 3 shows that the experimental cracking moments were higher than the predicted cracking moment by 0.5–11%. The accuracy of the prediction is due to the fact that the equation for predicting cracking moment only depends on one variable, i.e., the properties of concrete. On the other hand, the code was less accurate and more conservative in predicting ultimate moment of the beams. In FRP beams, the experimental ultimate moments were 13–30% higher than the predicated values. Since both the reinforcement and concrete affect the ultimate moment of the beams, the behavior of the beams is less predictable. Thus, the code is more conservative and there is a higher range of discrepancy between the predicted and experimental ultimate moment. Exposure had no systematic impact on the cracking or on the ultimate moments. As shown in Table 3, beams 2T8BU and 2T8BE have similar cracking moment values and the same can be said about 2T16BU and 2T16BE. The difference in the ultimate moment is due to the concrete.

### 3.2. Bond-Dependent Coefficient (k_b_)

The flexural design of FRP reinforced concrete beams is often controlled by serviceability limit states rather than strength requirements. Serviceability is governed by factors such as cracks width, which is affected by bar spacing and bond behavior. The bond behavior between FRP bars and concrete is quantified through bond-dependent coefficient (*k_b_*). The bond coefficient (*k_b_*) gives a numerical value for the bond between FRP bars and concrete with respect to the bond between steel bars and concrete. The bond behavior of steel bars and concrete is given the base value of *k_b_* = 1. *k_b_* values lower than one indicate a bond behavior superior to that of steel reinforced concrete and vice versa [1]. Due to its conservative nature, the ACI 440.1R code [1] assumes that FRP reinforced concrete have inferior bond behavior when compared to steel reinforced concrete. Although it was found, through extensive analysis, that *k_b_* values usually fall in the range of 0.6–1.72 (with an average of 1.1), the ACI 440.1R [1] committee insists that a *k_b_* value of 1.4 is to be used in serviceability governed FRP reinforced concrete design when not enough information is available. Other codes such as the CAN/CSA S6 [34] assume that FRP reinforced concrete have superior bond behavior than steel reinforced concrete where a *k_b_* value of 0.8 is recommended for serviceability design.

The experimental *k_b_* factor in this study was calculated in accordance with the ACI440.1R provision [1]. After crack widths were measured using crack transducers, the *k_b_* values were calculated and tabulated, as shown in Table 4. The average *k_b_* value was 0.61, which is near the lower end of the reported values in the ACI 440.1R code [1]. This shows that the 1.4 recommended by the ACI 440.1R code [1] is extremely conservative. The experimental *k_b_* value is closer to the *k_b_* = 0.8 recommended by the CAN/CSA S6 [34]. Exposure had no systematic impact on the *k_b_* value. Beam 2T8BU had a higher *k_b_* value than beam 2T8BE but beam 2T16BU had a lower *k_b_* value than beam 2T8BE. Therefore, the change cannot be attributed to exposure.

### 3.3. Crack Width and Propagation

Although crack width is an important factor in the serviceability limit state design, the FRP reinforced concrete design codes permit larger crack widths than the steel reinforced concrete codes do. The added leniency in crack width consideration can be attributed to the fact that FRP bars are not affected by corrosion. Thus, the permitted crack width can be up to 0.7 mm according to the CSA/CAN S6 [34].

**Figure 7:**
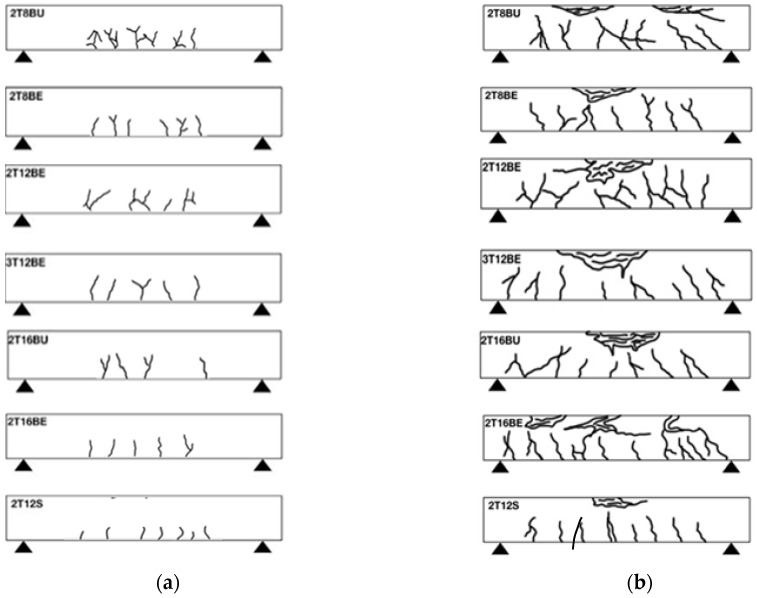
Crack pattern at: (**a**) service load; and (**b**) ultimate load.

The beams followed the typical flexural cracking behavior. After the concrete tensile strength was exceeded, longitudinal cracks began to appear in the constant moment zone. At the surface load, the cracks started to propagate from the tension zone to the compression zone beyond the neutral axis as shown in Figure 7a. Cracks become wider and more frequent the higher the load up until the crushing failure of concrete occurs at the ultimate load, as shown in Figure 7b. Exposure did not impact the crack pattern as can be seen for beams 2T8BU and 2T8BE and beams 2T16BU and 2T16BE.

**Figure 8:**
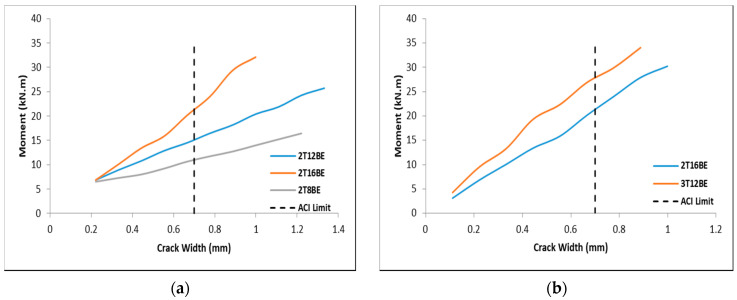
Moment vs. crack width: (**a**) different BFRP reinforcement ratios; and (**b**) different number of BFRP reinforcement bars.

The width of the cracks was impacted by the reinforcement ratio: higher reinforcement ratio leads to lower crack widths. In addition, reinforcement ratio directly influenced the stiffness of beams: stiffer beams lead to more cracks that are, however, smaller in width. As shown in Figure 8a, beam 2T16BE showed the most resistance to widening of cracks as higher moment is required to cause the same crack width when compared with, e.g., 2T12BE. Stiffness of the beam can be also caused by increasing the number of reinforcing bars while keeping the reinforcement ratio constant. Figure 8b shows that, although 3T12BE and 2T16BE had similar reinforcement ratio, 3T12BE showed more resistance to cracking due to the increase in the number of reinforcing BFRP bars.

**Figure 9:**
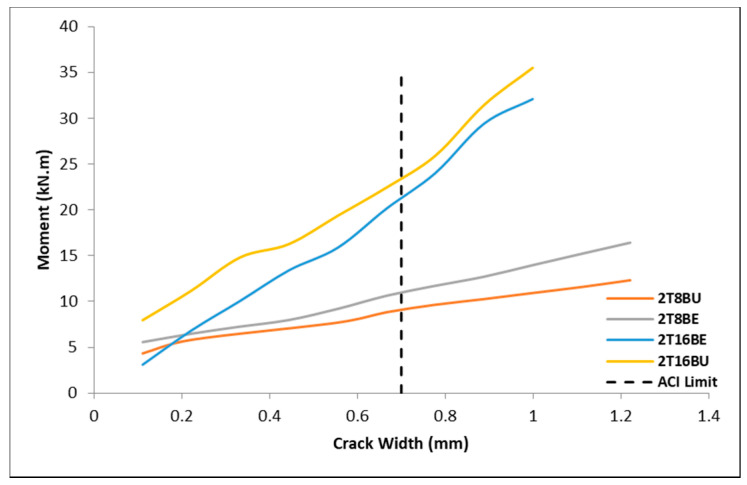
Moment vs. crack width of exposed vs. unexposed BFRP bars.

Figure 9 shows the effect of exposure on the crack width of the beams. Comparing the beams with similar reinforcement detailing but different exposure conditions, it can be seen in Figure 9 that the exposure had no systematic impact on the stiffness and crack widths of the beams. Particularly, beams 2T8BU and 2T8BE have comparable stiffness and similar trend in crack width propagation. Similarly, the exposure of BFRP bars in beam 2T16BE did not affect the stiffness and crack width of the beam compared to 2T16BU.

### 3.4. Flexural Capacity and Mode of Failure

**Figure 10:**
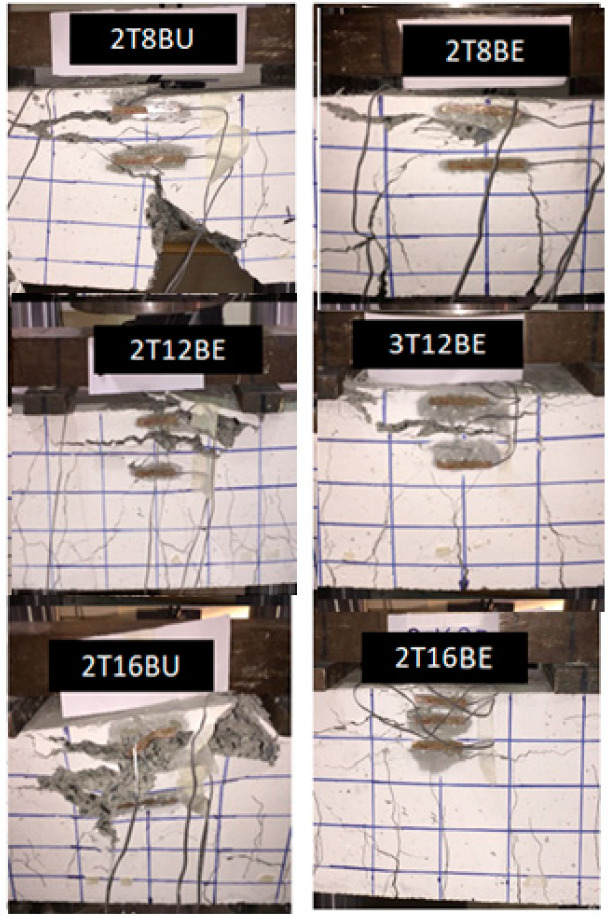
Modes of failure for BFRP reinforced beams.

All BFRP reinforced concrete beams were designed to fail by concrete crushing. The brittle nature of FRP material makes concrete crushing a less sudden type of failure. The concrete begins to weaken with the increased strain until it reaches its strain of 0.002, after which the section would fail. As shown in Figure 10, the concrete section failure was the controlling failure mechanism. After increased loading, the concrete section reached its maximum strain before failure. The failure occurred above the neutral axis in the compression zone and the FRP bars did not reach their ultimate strain as per the ACI 440.1R recommendations.

**Figure 11:**
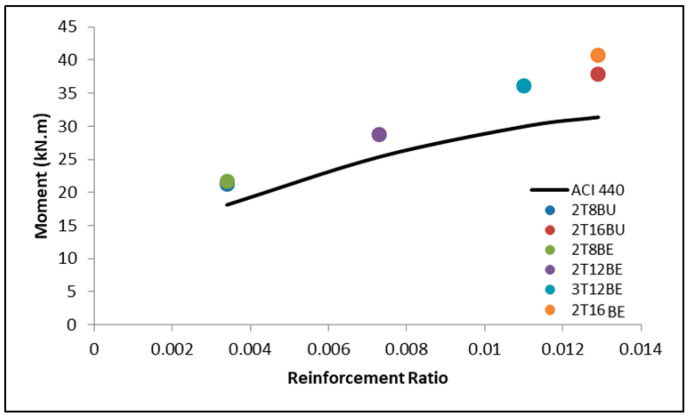
Ultimate moment vs. reinforcement ratio curve.

As stated above, the code is conservative in regards to predicting the ultimate moment of BFRP reinforced concrete beams. The conservative prediction is shown through the schematic graph in Figure 11. All six BFRP reinforced beams lay above the predicted moment values, regardless of the reinforcement ratio. Figure 11 also shows the impact of increasing reinforcement ratio on the ultimate moment. The impact of increasing the reinforcement ratio does not result in directly proportional increase in the ultimate moment, as shown in [11]. For example, when the reinforcement ratio of 0.0034 (2T8BE) is increased to 0.0073 (2T12BE), the 115% increase only yields a 32% increase in the ultimate moment capacity (from 21.7 to 28.7 kN·m). Furthermore, an extreme increase of 280% in reinforcement ratio (2T8BE to 2T16BE) yields 87% increase in moment capacity. Thus, it can be stated that the moment capacity increase resulting from increasing the reinforcement ratio, although significant, is not directly proportional but follows the same increasing trend of the ACI 440.1R [1] equation.

### 3.5. Moment–Deflection Behavior

**Figure 12:**
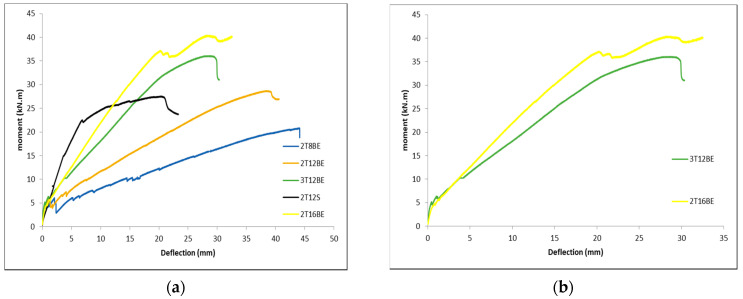
Moment vs. mid-span deflection: (**a**) different BFRP reinforcement ratios; and (**b**) different number of BFRP reinforcement bars.

**Figure 13:**
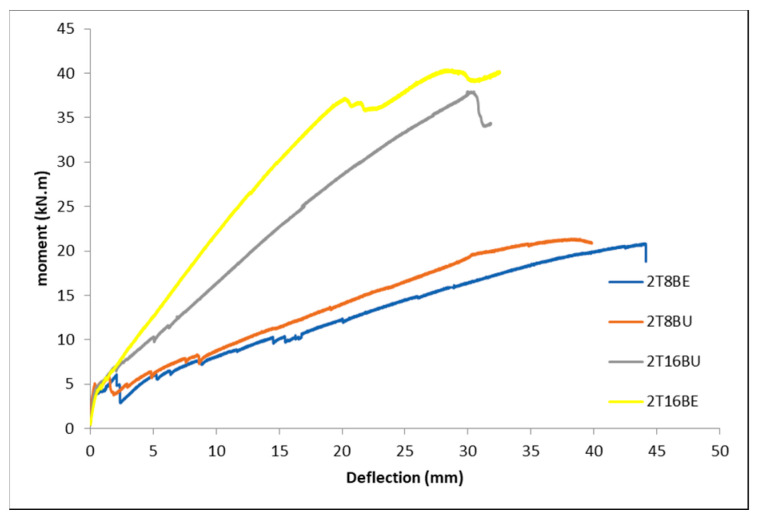
Moment vs. mid-span deflection of exposed vs. unexposed BFRP bars.

The moment versus mid-span deflection of all specimens in this study are shown in Figure 12 and Figure 13. Compared to control steel reinforced beam 2T12S, exposed BFRP reinforced specimen 2T12BE exhibited significantly lower stiffness but slightly higher ultimate moment capacity. Furthermore, beam 2T12BE failed at a considerably larger deflection than beam 2T12S. Figure 12 and Figure 13 clearly indicate that, prior to the first cracking moment, all beams follow the same moment vs. mid-span deflection trend. After the first crack, the stiffness of the beams plays a major role in determining the moment vs. mid-span deflection for the FRP reinforced beams. Stiffer beams yielded steeper (resulting in lower deflection values) slope and vice versa. As aforementioned, stiffness can be increased through increasing the reinforcement ratio or by increasing the number of bars. Figure 12a shows that the increase in reinforcement ratio yielded in stiffer beams that are more resistant toward deflection. This is reflected in the graphs of beams 2T8BE, 2T12BE, and 2T16BE. The “constant” axial stiffness mandate is not met between 3T12BE and 2T16BE since these specimens did not have equal stiffness (16.9 and 19.5 MN, respectively). The subtle difference in axial stiffness, while affecting the crack width as mentioned above, did not affect the behavior the beams in deflection. Although 3T12BE has more reinforcement bars, the slightly lower axial stiffness compered to 2T16BE makes the latter beam more resistant toward deflection, as shown in Figure 12b.

The exposure had no significant influence on the moment vs. mid-span deflection of the beams, as shown in Figure 13. In the 8-mm BFRP reinforcement, the unexposed bars reinforced beams show more resistance against deflection. The case is opposite for the 16-mm reinforcement.

### 3.6. Reinforcement and Concrete Strain

Table 5 summarizes the reinforcement and concrete strain values at ultimate moment. The strain behavior is similar in all beams before the first crack occurs; afterwards, the reinforcement ratio plays a major part in the magnitude of strain. As the reinforcement ratio increases, the strain in the reinforcement decreases. Beams with higher reinforcement ratios showed lower reinforcement strains. Specimens 2T16BE and 2T16BU (having the highest reinforcement ratio) yielded the lowest reinforcement strains, 0.0131 and 0.0128, respectively, as indicated in Table 5. The results also show that the effect of exposure on the BFRP reinforcement strains was insignificant (see the strain values for the two 2T8B beams and the two 2T16B beams in Table 5).

## 4. Finite Element Modeling (FEM)

Since the impact of exposure on over-reinforced BFRP RC beams was investigated experimentally in this paper, the main goal of the FE study was to investigate the effect of exposure on the strength and ductility of under-reinforced BFRP RC beams. As a result, a parametric study was conducted to investigate the effect of reduction in tensile strength of BFRP for under-reinforced beams (2T6B). The simulation was performed using commercial finite element software ABAQUS (Dassault Systèmes, RI, USA). To validate the model, four beams (2T8BU, 2T16BU, 2T12BE, and 3T12BE) were simulated and then validated with the experimental results. The mixture of unexposed and exposed beams simulation was done for control purposes. The geometric and materials nonlinearities were captured by incorporating the nonlinear geometry (*NLGEOM) formulation in the general static step in ABAQUS. In addition, analysis of all full three-dimensional beams was carried out under displacement-controlled loading mode. Post-processing included graphing moment–displacement responses; displacement values were obtained from maximum displacement at mid-span of the beams and load values were obtained from the summation of the reaction forces of the two supports.

### 4.1. Material Properties

**Figure 14:**
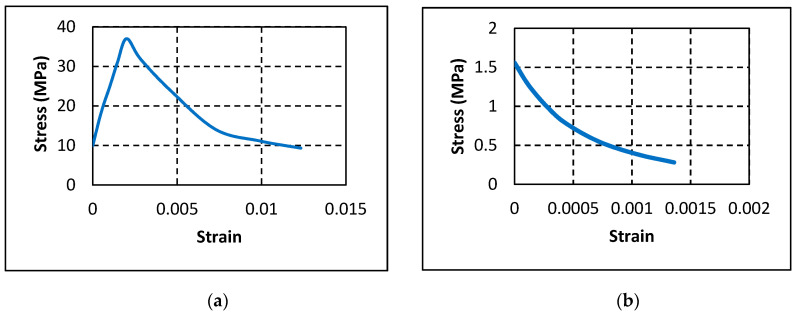
Concrete properties: (**a**) compressive stress–strain curve; and (**b**) tensile stress–strain curve.

The inelastic response of concrete was simulated using Concrete Damaged Plasticity (CDP) model incorporated in the software that applies the yielding equation proposed by Lubliner et al. [35] and later revised by Lee and Fenves [36]. Details of the parameters incorporated in the CDP model are shown in Table 6 as provided in ABAQUS user’s manual [37]. Figure 14a,b show the compressive and tensile stress–strain relationships of concrete as obtained from the experimental test results, respectively.

Steel used in top reinforcement and stirrups was defined to have an elastic modulus of 200 GPa and Poisson’s ratio of 0.3. The yield stress was defined to be 460 MPa. Unlike the ductile behavior of steel, BFRP bars have linear stress–strain behavior until rupture. To simulate this behavior in ABAQUS, the elastic modulus and Poisson’s ratio were defined as 49 GPa and 0.2, respectively.

### 4.2. Beam Geometry and Mesh Sensitivity

**Figure 15:**
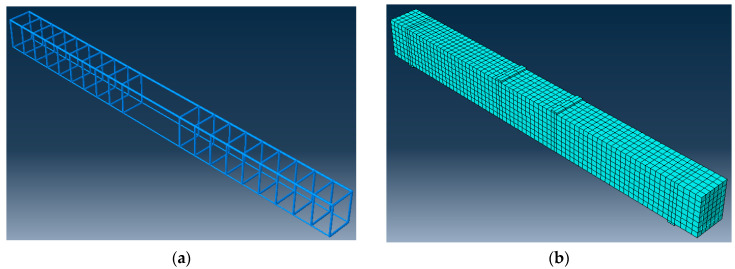
FEM model: (**a**) reinforcement cage; and (**b**) mesh configuration.

Concrete was modeled as eight-node linear 3D solid element with reduced integration (C3D8R). Steel and BFRP were modeled as two-node linear 3D truss elements (T3D2) that are embedded in the concrete by introducing an embedded constraint and defining steel and BFRP bars as embedded region and concrete part as host region. A mesh size of 30 mm was chosen based on a mesh sensitivity study performed to select the optimum mesh size that simulates the experimental results with minimum computational time. Figure 15a,b shows the reinforcement cage for a beam with 2 BFRP bars and mesh configuration, respectively.

### 4.3. Model Verification

**Figure 16:**
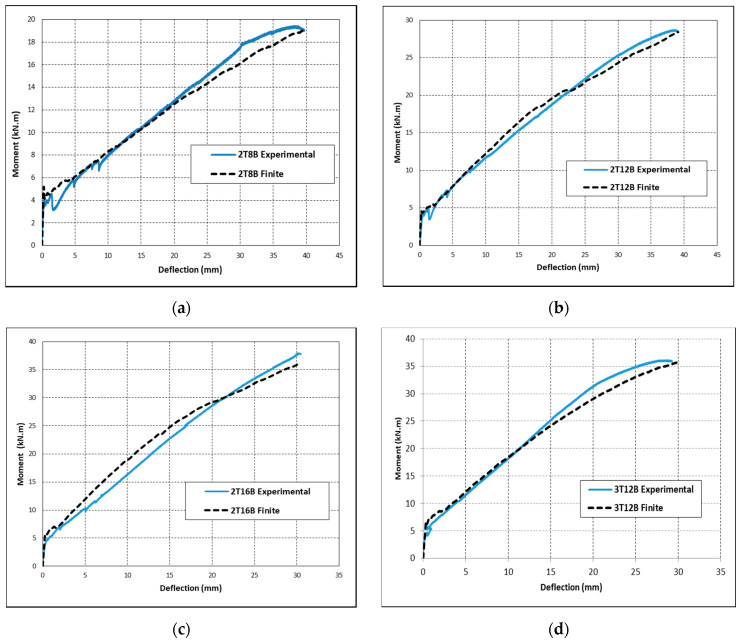
Model verification for beams: (**a**) 2T8B; (**b**) 2T12B; (**c**) 2T16B; and (**d**) 3T12B.

**Figure 17:**
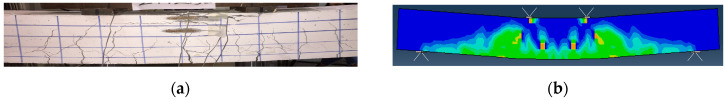
Crack distribution for beam 2T12B at failure: (**a**) experimental; and (**b**) deformed shape of FE model (PEEQT).

Validation of the simulated FE models was conducted by comparing moment vs. mid-span deflection responses of the FE generated curves with the experimental results. Figure 16 shows the tested and simulated moment–displacement curves for beams 2T8B, 2T12B, 2T16B, and 3T12B. Figure 16 shows that all simulated results are in good agreement with the experimental results at all stages of loading until failure. Mainly, the moment at first crack, initial stiffness, and ultimate capacity of the beams of the FE models aligns with the experimental curves. In addition, Figure 17 shows the crack pattern observed experimentally as opposed to the crack pattern shown in ABAQUS for beam 2T12B. It can be noticed in Figure 17 that crack distribution along the beam length was accurately captured by the FE model.

The accuracy of the FE models was also validated by comparing the flexural capacity and maximum mid-span deflection of the numerical results with the experimental results. Table 7 shows the results in terms of the experimental ultimate moment (*M_EXP_*), numerical ultimate moment (*M_FE_*), experimental-to-predicted ratio of ultimate moment (*M_EXP_/M_FE_*), experimental maximum mid-span deflection (Δ*_EXP_*), numerical mid-span deflection (Δ*_FE_*), and experimental-to-predicted mid-span deflection (Δ*_EXP_/*Δ*_FE_*). It is clear in Table 7 that the results of the simulated models agree with the experimental values. In particular, the average *M_EXP_/M_FE_* is 1.02 with a standard deviation of 0.03 and the average Δ_EXP_/Δ_FE_ is 1.00 with standard deviation of 0.02.

### 4.4. Parametric Study

The verified models were utilized to carry out the parametric studies for the reduction of tensile strength in on 2T6B beam. The objective of the parametric study was to simulate the effect of exposure of BFRP bars on the capacity and ductility of under-reinforced BFRP RC beams. The impact of exposure on over-reinforced beams does not affect the flexural behavior of beams; it only affects the serviceability behavior of said beams. However, in under-reinforced beams, the impact of exposure (reduction in tensile strength) does affect the flexural behavior of the beam. Therefore, the tensile strength of the under-reinforced beam 2T6B was reduced by 10%, 20%, and 30% to show the impact of potential exposure of these beams. Table 8 shows the values of the ultimate moment (*M*) and maximum mid-span deflection (Δ) obtained from the numerical models for all reduction groups.

#### Effect of Reduction in the Tensile Strength

**Figure 18:**
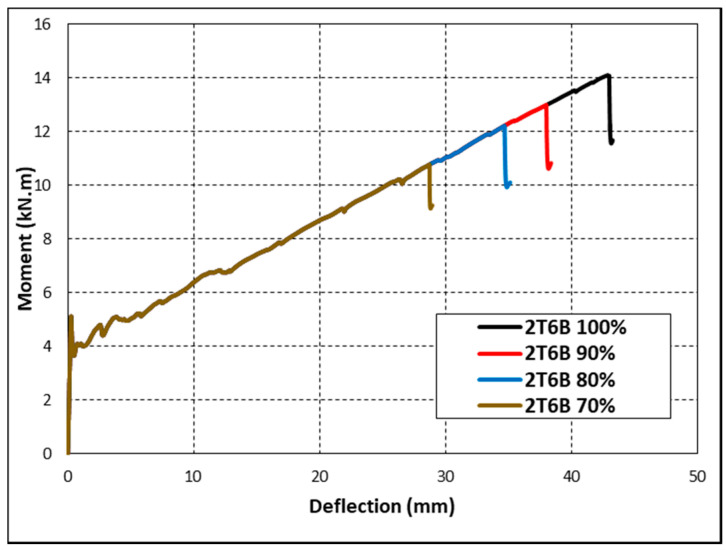
Parametric test moment–deflection curves for beam 2T6B.

Figure 18 presents the moment versus mid-span deflection behavior of the FE models examining the effect of reduction of the tensile strength of BFRP on the flexural behavior of 2T6B (under- reinforced) beam. Figure 18 and Table 8 show that the reduction in tensile strength caused a disproportional decrease in the flexural capacity of the beam. A reduction of 10% reduced the beam’s capacity by 7.8%, while a reduction of 30% reduced the beam’s capacity by 23.5%. However, a clear direct proportional trend was observed between the maximum deflection and tensile strength. For example, reducing the tensile strength by 10% decreased the deflection by 11.52%. Likewise, a decrease in tensile strength by 20% and 30% decreased the deflection by 19.38% and 33.07%, respectively. The same reduction in over-reinforced beams (tested experimentally) would not have yielded similar results. In fact, it is possible that the reduction in tensile strength presented in this section would not affect the behavior of the beams studied in the previous sections at all, that is unless the over-reinforced beams become under-reinforced beams due to the tensile reduction.

## 5. Conclusions

The paper aims to add to the body of knowledge in the area of BFRP reinforced concrete beams. The effects of environmental (site-like) exposure on the serviceability and flexural behavior of over-reinforced BFRP RC beams were investigated. In addition, a finite element molding was used to create a parametric study to predict the effects of reduction in tensile strength (caused by exposure) on the flexural behavior of under-reinforced BFRP RC beams. The experimental program involved casting and testing seven beams, four of which were casted using exposed BFRP Bars, two with unexposed BFRP bar, and one reinforced with steel for control purposes. The flexural tests were conducted using a four-point loading test. The following conclusions can be drawn from this investigation:

- The experimental cracking moments were higher than the predicted cracking moment by 0.5–10.5% while the experimental ultimate moments were higher than the predicted cracking moment by 12–30%. The ACI 440.1R code is more conservative when predicting ultimate moment than when predicting cracking moment. The long-term exposure did not show significant impact on the cracking or on the ultimate moments.
- The average *k_b_* value was 0.61 for both exposed and unexposed BFRP bars, which is near the lower end of the reported values in the ACI 440.1R code and show that the 1.4 recommended by the said code is extremely conservative. On the other hand, the experimental *k_b_* value was closer to the *k_b_* = 0.8 recommended by the CAN/CSA S6.
- Increasing reinforcement ratio and increasing the number of BFRP reinforcing bars led to an increase in the stiffness of BFRP reinforced concrete beams. Stiffer BFRP reinforced beams showed more resistance toward cracking. In stiffer beams, the number of cracks increased, which, in turn, decreased the spacing between said cracks and reduced the widths of cracks. The crack width did not get impacted by the presence or lack of exposed reinforcing bars.
- For both exposed and unexposed BFRP RC beams, increasing the reinforcement ratio did not result in a directly proportional increase in the moment capacity. However, the increasing trend of the moment capacity for the BFRP RC beams was similar to the ACI 440 theoretical equation.
- Exposure did not have systematic impact on the moment vs. mid-span deflection behavior of the BFRP reinforced concrete beams. Beams with higher reinforcement ratios exhibited lower reinforcement strains.
- Exposure did not have systematic impact on the strain in the BFRP reinforcement at all. The strain in reinforcement was in close proximity for all BFRP beams with similar reinforcement ratio regardless of exposure.
- The FEM parametric study showed that reduction in the tensile strength of BFRP bars in under-reinforced BFRP RC beams caused a disproportional decrease in the flexural capacity of BFRP RC beams.

The findings of this study can only be generalized for BFRP bars of similar properties as that of Galen. Furthermore, the response to exposure can only be generalized to environments with hot weather. There needs to be further research on BFRP bars from different manufacturers and further research on the impact of cold weather on these bars.

## Figures and Tables

**Table 1:** Mechanical properties of reinforcing bars.

Bar Type	Diameter (mm)	Area (mm^2^)	Ultimate Tensile Strength (MPa)	Yield Stress (MPa)	Elastic Modulus (GPa)
BFRP	8	57.4	1121.3 ± 56	-	42.9 ± 1.4
	12	121.3	1118.6 ± 31	-	46.6 ± 1.7
	16	211.9	1075.1 ± 37	-	46.0 ± 2.1
Steel	10	78.5	-	460	200
	12	113.0	-	460	200

**Table 2:** Test matrix.

Beam No.	Beam ID	Bar Size (mm)	Area (mm^2^)	Type of Reinforcement	*ρ_f_*	*ρ_f_*/*ρ_fb_*	Axial Stiffness EA (MN)
1	2T12S	12	226.0	Steel	0.0068	0.18	45.2
2	2T8BU	8	114.5	BFRP-Unexposed	0.0034	1.61	4.9
3	2T8BE	8	114.5	BFRP-Exposed	0.0034	1.61	4.9
4	2T12BE	12	243.0	BFRP-Exposed	0.0073	3.47	11.3
5	3T12BE	12	364.5	BFRP-Exposed	0.0110	5.21	16.9
6	2T16BU	16	424.0	BFRP-Unexposed	0.0129	6.20	19.5
7	2T16BE	16	424.0	BFRP-Exposed	0.0129	6.20	19.5

**Table 3:** Experimental vs. predicted ultimate and cracking moments.

Beam	Experimental			Exp./Pred. ACI [1]		Failure Mode
	*M_n_* (KN.m)	δ (mm)	*M_cr_* (kN.m)	*M_n_*	*M_cr_*	
2T12S	27.05	20.70	6.35	1.49	1.07	TC ^1^
2T8BU	21.30	39.69	5.96	1.18	1.01	CC ^2^
2T8BE	21.70	44.10	6.56	1.20	1.11	CC
2T12BE	28.70	39.00	6.06	1.13	1.03	CC
3T12BE	36.10	29.27	6.17	1.20	1.04	CC
2T16BU	37.90	30.48	6.02	1.21	1.02	CC
2T16BE	40.70	29.30	6.45	1.30	1.09	CC
Average	-	-	-	1.25	1.05	-

^1^ TC, tension control (steel yielding); ^2^ CC, concrete crushing.

**Table 4:** Experimental bond-dependent coefficient (*k_b_*).

Beam	0.30*M_n_*	w = 0.7 mm	Average
2T8BU	0.48	0.70	0.59
2T8BE	0.22	0.57	0.40
2T12BE	0.64	0.78	0.71
3T12BE	0.50	0.47	0.49
2T16BU	0.48	0.69	0.59
2T16BE	0.94	0.89	0.92
Average	0.54	0.68	0.61

**Table 5:** Reinforcement and concrete strain at ultimate moment.

Beam	Moment (kN·m)	Longitudinal Reinforcement Strain	Concrete Strain
2T12S	27.05	0.0323	0.00223
2T8BU	21.3	0.0233	0.00282
2T8BE	21.7	0.0228	0.00214
2T12BE	28.7	0.0145	0.00292
3T12BE	36.1	0.0137	0.00315
2T16BU	37.9	0.0128	0.00209
2T16BE	40.7	0.0131	0.00316

**Table 6:** Concrete damaged plasticity parameters.

Dilation Angle	Eccentricity	fb0a ^1^/fc0b ^2^	Kc ^3^	Viscosity
36.5	0.1	1.16	0.667	0.00001

^1^ fb0, initial biaxial compressive strength of concrete.^2^ fc0, initial uniaxial compressive strength of concrete. ^3^ Kc, ratio of the second stress invariant on the tensile meridian to compressive meridian at initial yield.

**Table 7:** Comparison between experimental and predicted capacities and mid-span deflections.

Beam	*M_EXP_* (kN.m)	*M_FE_* (kN.m)	*M_EXP_/M_FE_*	Δ*_EXP_* (mm)	Δ*_FE_* (mm)	Δ*_EXP_/*Δ*_FE_*
2T8B	19.45	19.66	0.99	39.69	39.47	1.01
2T12B	28.72	28.42	1.01	39.00	39.10	1.00
2T16B	37.78	35.67	1.06	30.48	29.75	1.02
3T12B	36.12	35.89	1.01	29.27	30.18	0.97

**Table 8:** Parametric study results.

Beam	Tensile Strength (MPa)	Reduction	*M* (kN·m)	Δ (mm)
2T6B	1200	0%	14.08	42.88
2T6B	1080	10%	12.98	37.94
2T6B	960	20%	12.19	34.57
2T6B	840	30%	10.77	28.70
